# Neuromyelitis Optica: An Antibody-Mediated Disorder of the Central Nervous System

**DOI:** 10.1155/2012/460825

**Published:** 2012-01-29

**Authors:** Jiwon Oh, Michael Levy

**Affiliations:** Department of Neurology, Johns Hopkins University School of Medicine, 600 N. Wolfe Street, Pathology 509, Baltimore, MD 21287, USA

## Abstract

Neuromyelitis optica (NMO) is a recurrent inflammatory disease that preferentially targets the optic nerves and spinal cord leading to blindness and paralysis. The hallmarks of NMO include bilateral optic neuritis and longitudinally extensive transverse myelitis. Woman and African Americans are overrepresented in the US patient population. NMO is associated with the NMO-IgG biomarker, which targets the aquaporin-4 water channel on astrocytes. The humoral pathology of NMO lesions include IgG and IgM deposits and infiltration by granulocytes suggesting that the NMO-IgG may be involved in the pathogenesis of disease. This review of the recent NMO literature covers the clinical features, epidemiology, radiology and pathology of disease and includes discussion of the important basic science research work in the field.

## 1. Introduction

Neuromyelitis optica (NMO) is an autoimmune inflammatory disorder of the central nervous system that predominately affects the optic nerves and spinal cord.

In 1870, Allbutt was the first to report a case of NMO [1], but it was Devic who described the disorder in detail, and summarized 16 cases in the existing literature in 1894 [2]. Based on this initial description, historically, NMO has been regarded as a severe, generally monophasic disorder of the optic nerves and spinal cord and was thought to be a variant of multiple sclerosis (MS). A convincing body of evidence in the past decade has established NMO as a distinct disease entity from MS. NMO is now recognized as a recurrent disease that largely targets the spinal cord and optic nerves but can also affect the brain as well. NMO occupies a unique position in the spectrum of inflammatory central nervous system demyelinating disorders in that it is the only such disorder that has an associated disease-specific antibody, aquaporin-4 antibody (AQP4 Ab), or NMO-IgG. Recognition of this antibody has been instrumental in elucidating the underlying pathobiology and in guiding treatment options for NMO.

## 2. Clinical Features

The clinical hallmarks of NMO are acute optic neuritis that is often bilateral and transverse myelitis that is often longitudinally extensive. Commonly reported symptoms include unilateral and bilateral loss of visual acuity, ocular pain, severe paraplegia, a symmetric sensory level, bladder dysfunction, paroxysmal tonic spasms of the trunk and limbs, and Lhermitte's phenomenon [3, 4]. Rostral extension of cervical cord lesions into the cervicomedullary junction can cause symptoms such as acute respiratory decompensation, nausea, intractable vomiting, and hiccups. These symptoms can precede or occur in association with the more typical features of optic neuritis or transverse myelitis [3, 5–7]. 

Clinical features attributable to locations outside of the optic nerves and spinal cord can also occur in patients with NMO. Hypothalamic-pituitary axis dysfunction can manifest as hypersomnolence, hyponatremia, hypothermia, hypothyroidism, and hyperprolactinemia [8]. In addition, confusion, abrupt changes in level of consciousness, cortical blindness, and imaging findings suggestive of posterior reversible encephalopathy syndrome (PRES) have also been reported [9]. 

The clinical course of NMO historically took one of two forms: monophasic or relapsing, with relapsing forms comprising approximately 80–90% of cases. However, after an index event, the distinction between monophasic and relapsing NMO is often difficult to make since relapses can occur many years after an event. In the vast majority of cases (~80%), a relapse occurs by 2-3 years after the index event [3, 10]. Clinical features that may predict a relapsing course of disease include older age, female gender, less severe motor impairment with the initial myelitis event, and evidence of systemic autoimmunity [3]. 

Clinical attacks typically progress over days, with varying degrees of recovery seen in the ensuing weeks to months. Recovery is usually incomplete, and most patients sustain residual disability, which increases with subsequent attacks [3]. Factors predictive of mortality in patients with relapsing NMO include the presence of other systemic autoimmune disorders, higher attack frequency in the first two years, and poor motor recovery following the index myelitis event [10, 11]. Longitudinal case series of NMO patents with follow-up ranging from 5 to 10 years have demonstrated that the majority of patients (47–100%) have significant ambulatory difficulties at follow-up. Residual visual deficits are also common, with >60% of patients reporting significant vision loss in at least one eye. Mortality due to respiratory failure has been reported to take place in up to 32% of patients [12, 13]. Of note, this mortality figure was derived from the original Mayo Clinic study [3], which took place prior to the widespread recognition of NMO and NMOSDs, and the patient population may have been biased with respect to clinical disease severity. Therefore, the prognosis of NMO may not be as grave as was reported in these earlier studies.

In 1999, Wingerchuk et al. proposed diagnostic criteria for NMO which were based on clinical and radiographic features [3]. With the discovery of AQP4-Ab, these criteria were revised in 2006 to include the testing of this disease-specific antibody. In addition, the necessary clinical features included were modified and simplified in an attempt to improve the diagnostic properties of the criteria. At present, the 2006 proposed diagnostic criteria for NMO consist of the presence of optic neuritis and transverse myelitis as well as 2 out of 3 of a contiguous spinal cord MRI lesion extending over more than 3 vertebral segments (i.e., longitudinally extensive), brain MRI not meeting diagnostic criteria for MS, and NMO-IgG seropositive status [14]. These criteria are 99% sensitive and 90% specific for the diagnosis of NMO and have been independently validated in different patient populations [15]. 

The recent literature suggests that in addition to its utility in the diagnosis of NMO, the presence of NMO-IgG may have a role in disease prognosis. In a prospective study of patients with longitudinally extensive transverse myelitis (LETM), 55% of those positive for NMO-IgG relapsed with recurrent LETM or optic neuritis, while none of the seronegative patients relapsed [4]. Similarly, in a series of patients with recurrent optic neuritis, the presence of NMO-IgG heralded a 50% chance of developing transverse myelitis [16], while only 6.6% of seronegative patients developed transverse myelitis. More recently, Jarius et al. found that in acute monosymptomatic optic neuritis, 50% of NMO-IgG seropositive patients progressed to NMO within 12 months, while none of the seronegative patients progressed after a median follow-up of 26 months [17]. 

In light of the fact that NMO is a disorder that has the potential to cause significant disability, the ability to recognize and differentiate NMO and related disorders from other demyelinating disorders is important from a clinical perspective. The term “NMO spectrum disorders” has been coined to reflect a variety of disorders thought to be related to NMO but do not quite meet the clinical diagnostic criteria for definite NMO. Disorders that are typically included in this classification are NMO-IgG seropositive limited forms of NMO (single or recurrent LETM, recurrent or simultaneous bilateral ON), Asian opticospinal MS (OSMS), optic neuritis or LETM associated with systemic autoimmune disease, and optic neuritis or myelitis associated with brain lesions typical of NMO (e.g., hypothalamic or brainstem lesions) [12]. Whether the NMO-IgG seronegative forms of these disorders are a forme fruste of classic NMO or whether they are variants of other autoimmune diseases is, at present, unclear. Until we are able to better identify with certainty that these are distinct disorders, the designation of NMO spectrum disorders is useful, as it has specific prognostic and therapeutic implications for these potentially related disorders.

## 3. Epidemiology

NMO has a distinct epidemiological profile in comparison to MS. The median age of onset of NMO is typically in the fourth or fifth decade, which is older than the average age of onset of MS. The age of onset can be quite variable, however, and NMO is occasionally seen in children and the elderly. There is a significant female predominance in both diseases, but it is even more polarized in NMO, with a female-to-male ratio ranging from 5–11 : 1 [3, 10, 13, 18]. 

From a global perspective, NMO occurs much more commonly in nations with a predominately nonwhite population make-up, where it is a common cause of CNS demyelination. In Japan, up to 15–40% of MS is comprised of the opticospinal variant, which may be a synonymous disorder with NMO [19]. Lau et al. reported that up to 36% of MS cases in Hong Kong had selective involvement of the optic nerves and spinal cord [20], and NMO comprised 17% of possible MS cases in French Afro-Caribbeans in Martinique [21]. In a population-based study in Cuba, NMO comprised approximately 10% of demyelinating disorders [22]. In contrast, in countries consisting of a predominately white population, NMO comprised less than 2% of all demyelinating disorders, and the majority of cases occurred in white patients [23]. Similarly, in the Mayo series, Wingerchuk et al. found that NMO still tends to occur predominately in white populations [3]. 

Our experience at the NMO clinic at Johns Hopkins Medical Institution has shown a significant racial predilection for NMO with African American populations comprising approximately 50% of patients, which is clearly epidemiologically distinct from MS (unpublished data).

Of note, there is a differential demographic profile in monophasic and relapsing NMO, with a stronger female predominance in relapsing NMO (female : male = 9 : 1), while monophasic NMO seems to equally affect both genders (female : male = 1 : 1). In addition, the median age of onset in monophasic NMO is a decade earlier than relapsing NMO (29 years versus 39 years, resp.) [3]. 

## 4. Genetics

The distinct racial predilection of NMO suggests a possible genetic etiological contribution. Although there have been a handful of reports on familial cases of NMO, to date, a convincing human leukocyte antigen (HLA) class II allele association has not been established [24–27]. 

## 5. Radiological Features

### 5.1. Conventional Imaging Techniques

**Figure 1:**
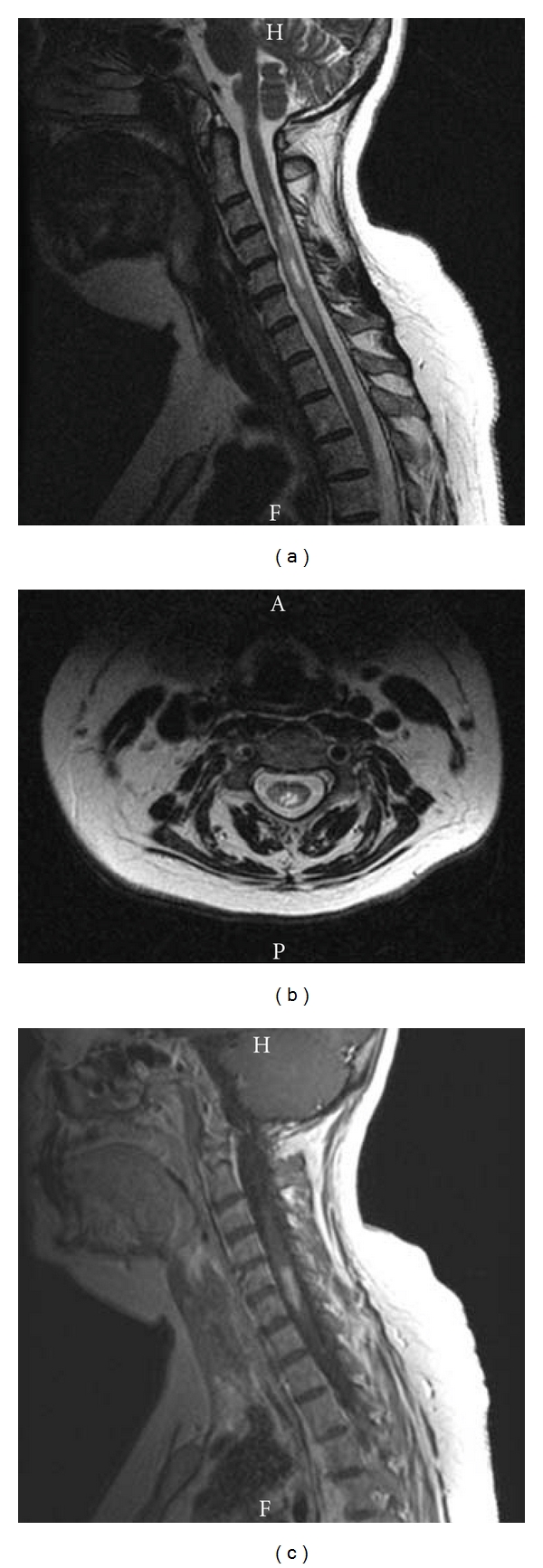
An MRI showing a longitudinally extensive cervical spinal cord lesion in a patient with NMO. (a) T2 weighted sagittal MRI sequence shows T2-hyperintensity extending beyond 3 spinal cord levels. (b) T2-weighted axial sequence shows bilateral T2 hyperintensity in the central/dorsal cord. (c) T1-weighted sagittal sequence with gadolinium contrast shows enhancement in a significant portion of the lesion.

Conventional MRI is an important tool in the diagnosis of NMO. A classic MRI feature that is seen in the majority of NMO patients is a longitudinally extensive transverse myelitis (LETM) that spans more than 3 vertebral levels, which is associated with cord swelling and gadolinium enhancement in many cases (Figure 1). The extent of the longitudinally extensive lesion in the cord, however, can be related to the proximity to the attack; therefore, in some cases the absence of LETM does not necessarily exclude the diagnosis of NMO. Cord atrophy can be observed in 22% of cases with follow-up imaging. Enhancement along the course of the optic nerve is another typical finding if the MRI is performed in close temporal proximity (within two weeks) of the onset of symptoms of optic neuritis [3]. 

 Although a “normal” MRI of the brain was initially thought to be a criterion of NMO, subsequent investigations confirm that the MRI brain is, in fact, abnormal in the majority of NMO patients. In patients with clinical and radiological features otherwise typical for NMO, 60–85% of cases have been shown to have abnormal brain lesions. Over time, up to 10% of patients develop lesions typical of MS [28, 29].

 Lesions involving the diencephalon and brainstem distinctly atypical for MS have been commonly reported in NMO patients [8, 29, 30]. These distinctive lesions predominately involve the hypothalamus and can extend to brain tissue surrounding the third and fourth ventricle and aqueduct of Sylvius and seem to be characteristic brain lesions of NMO. In a subsequent study, Pittock et al. demonstrated that these regions correspond with areas of AQP4 channel localization, which supports the notion that this area is particularly sensitive to pathogenic NMO-IgG [31]. 

The revised diagnostic criteria for NMO recognize the common brain abnormalities seen in NMO patients and were modified to include patients with abnormal brain MRI atypical for MS, rather than those with normal brain MRI [14]. 

### 5.2. Advanced Imaging Techniques

Advanced MRI techniques have enabled the establishment of further distinctions between NMO and MS patients, which increasingly supports the notion that NMO is a distinct disease entity. Filippi et al. demonstrated normal magnetization-transfer ratio (MTR) in the normal-appearing brain tissue of NMO patients, while patients with MS showed a significantly decreased MTR. In the spinal cord, on the other hand, MTR was significantly diminished in both NMO and MS patients. These findings correspond with clinical observations and suggest that the brain lesions of NMO are less destructive and, therefore more commonly asymptomatic, while spinal cord lesions tend to be much more destructive, and, therefore, more clinically symptomatic [32]. A subsequent study using both diffusion-tensor and magnetization-transfer imaging of the brain assessed normal-appearing gray and white matter separately in NMO and demonstrated no abnormalities in normal-appearing white matter, but diminished MTR and increased mean diffusivity (MD) in the normal-appearing gray matter, suggesting the presence of tissue disruption in the gray matter of NMO patients [33]. 

More recently, Yu et al. demonstrated the presence of abnormal diffusion (lower fractional anisotropy and higher mean diffusivity) in the white matter and gray matter of NMO patients in comparison to controls. Interestingly, further analysis of the white matter abnormalities showed that regions connected to the optic nerves and spinal cord had abnormal diffusion, while regions unconnected to the optic nerves and spinal cord demonstrated normal diffusion. This finding suggests that the observed abnormalities in water diffusion of the white matter tracts in NMO are largely caused by secondary degeneration from primary lesions in the optic nerves and spinal cord, rather than independent lesions [34]. A more recent study investigating the normal-appearing white matter in the spinal cord of NMO patients confirmed the presence of abnormal diffusion (diminished fractional anisotropy, elevated mean, and perpendicular diffusivity) in normal-appearing white matter and demonstrated a significant correlation of diffusion-tensor imaging-derived indices to global measures of clinical dysfunction. Based on these findings, the authors postulated that the pathological substrate of clinical dysfunction in NMO was more likely due to demyelination rather than axonal loss and that diffusion-tensor imaging-based indices may be useful as biomarkers for NMO [35]. 

### 5.3. Optical Coherence Tomography

Optical coherence tomography is an increasingly utilized method to visualize retinal pathology in optic-nerve-related disorders. de Seze et al. demonstrated a significantly thinner retinal nerve fiber layer (RNFL) in NMO patients in comparison to controls and a significant correlation to EDSS, a global measure of disability [36]. Subsequent reports comparing NMO and MS patients found that the RNFL in NMO patients is thinner on average, and that, after an episode of optic neuritis, the RNFL is significantly thinner in NMO patients, suggesting more substantial retinal damage following optic neuritis in NMO in comparison to MS [37, 38]. 

Taken together, these findings confirm the utility of novel imaging techniques in providing increasing insight into NMO disease mechanisms.

## 6. Laboratory Findings

Cerebrospinal fluid (CSF) analysis in NMO shows nonspecific abnormalities in the majority of cases. Pleocytosis is observed in 30–79% of patients with an acute exacerbation [3, 10, 13], with approximately 13–35% showing >50 cells/mm^3^, which is an uncommon finding in MS [3, 10, 18]. A neutrophilic pleocytosis is found in 17–57% of patients [3, 13], and oligoclonal bands are present in 17–43% of patients [3, 10, 13, 18]. CSF protein is elevated in 25–30% of patients [10, 18].

Laboratory evidence of non-organ-specific systemic autoimmunity is also common, and up to 25–44% of patients with or without clinical evidence of a systemic autoimmune disorder have a positive antinuclear antibody [3, 13, 18, 39]. Other non-organ-specific autoantibodies (SSA, SSB) are also commonly seen in NMO patients, and systemic autoimmune disorders such as Sjogren's Syndrome (SS) and systemic lupus erythematous (SLE) can occur concomitantly with NMO, which likely reflects an underlying predisposition for these patients to develop autoimmune disorders. A study by Pittock et al. in 2008 demonstrated the utility of NMO-IgG in distinguishing patients with concurrent NMO spectrum disorders and a systemic autoimmune disease from those with a neurological complication of systemic autoimmune disease. Patients with SS or SLE who were NMO-IgG positive were likely to have a history of transverse myelitis or optic neuritis, which suggests the coexistence of a NMO spectrum disorder with their systemic autoimmune disorder. On the other hand, patients seronegative for NMO-IgG did not develop optic neuritis or transverse myelitis, confirming the specificity of this antibody in detecting NMO and suggesting that the presence of NMO-IgG is not an epiphenomenon of general systemic autoimmunity [39].

## 7. Anti-Aquaporin-4 Antibody (NMO-IgG)

The discovery of the NMO-IgG has played a pivotal role in the diagnosis and elucidation of disease mechanisms in NMO.

The discovery of an NMO disease-specific antibody (NMO-IgG) by Lennon et al. in 2004 was fuelled by the observation that immunoglobulin and complement deposition in active lesions followed a distinct rim and rosette vasculocentric pattern, suggesting an antibody-mediated mechanism of disease [40]. Later, the target of NMO-IgG was identified to be the aquaporin-4 (AQP4) water channel [41].

The sensitivity and specificity of the NMO-IgG in identifying NMO in the Lennon et al. series were 73% and 91%, respectively [40]. Subsequently, multiple groups have validated the specificity of the NMO-IgG in identifying NMO using various immune assays. Waters and Vincent compared the sensitivities and specificities of the five main methods developed in relapsing NMO patients, which include indirect immunofluorescence (IIF), a cell-based assay (CBA), a radio-immunoprecipitation assay (RIPA), a fluoroimmunopireciptation assay (FIPA), and enzyme-linked immunosorbent assay. All techniques had high specificities, ranging from 91 to 100%, with the CBA and FIPA both having specificities of 100%. Sensitivities, on the other hand, varied and ranged from 57 to 91%, with IIF, FIPA, and CBA showing the highest sensitivities at 86, 83, and 91%, respectively [42].

There is evidence that rates of NMO-IgG seropositivity in patients meeting clinical diagnostic criteria for NMO may vary significantly depending on the population being tested. A study comparing NMO-IgG seropositivity in patients fulfilling the 1999 Wingerchuk diagnostic criteria for NMO in the Caribbean and Europe demonstrated a lower rate in the Caribbean (33.3%), as compared with Caucasian patients from Spain and Italy (62.5%) and France (53.8%) [43]. In comparison, 2 studies of NMO-IgG detection rates in Japanese patients with OSMS and LETM varied significantly, with one reporting NMO-IgG seropositivity in 60% of patients, [44] while another reports seropositivity in 35% of such patients [45].

At present, it is unclear whether there is truly a subset of patients with clinical NMO that are NMO-IgG negative, or if this is a result of inadequate sensitivity of existing immunoassays to detect the antibody, or inadequately sensitive and specific diagnostic criteria, or a combination of all of these factors. Given the variability in the diagnostic properties of different assays, until further insight is available into this issue, current expert recommendations are to test for the presence of NMO-IgG using two separate detection methods, one of which should be indirect immunofluorescence [46, 47].

## 8. Pathological Features and Proposed Disease Mechanisms

AQP4 is an integral homotetrameric protein complex and is expressed primarily on the abluminal surface of astrocyte foot process and can also be found on ependymocytes and endothelial cells [48]. It is one of the main water channels in the central nervous system but is also found outside of the central nervous system in many solid organs including renal distal collecting tubules and in a portion of gastric parietal cells. In astrocytes, AQP4 is anchored by a dystroglycan complex [49] and is found in highest concentrations in astrocytes having direct contact with capillaries and pia in the brain and spinal cord [50]. AQP4 likely acts in concert with potassium and bicarbonate channels to regulate water dynamics in the CNS between brain, blood, and CSF and is therefore an integral element of brain volume and ion homeostasis [48, 49]. AQP4 has also been shown to play a role in astrocyte migration and neural signal transduction in animal models [51]. The absence of AQP4 in knockout mouse models is known to exacerbate the recovery from vasogenic edema, and AQP4 has been shown to mediate cerebral edema in various models of neurological injury, including epilepsy, ischemia, and trauma [52, 53]. Interestingly, a recent study in AQP4-knockout mice found that experimental autoimmune encephalomyelitis (EAE) was attenuated in comparison to wild-type mice, which implicates AQP4 as a novel disease determinant of the severity of EAE and likely other autoimmune disorders of the CNS [54].

Zhang and Verkman demonstrated the presence of high concentrations of AQP4 in perivascular astrocytic foot processes in the cerebral cortex, subependymal and vasculocentric regions of the brainstem, and gray and white matter of the spinal cord, and in a vasculocentric pattern in the optic nerves [55]. This pattern seems to coincide, for the most part, with sites of preferential lesion formation in NMO. However, the presence of high concentrations of AQP4 in astrocytic foot processes in the cerebral cortex, as well as in retinal Muller cells [48], which are not areas in which NMO lesions are typically seen clinically, suggests that there is selectivity of AQP4 involvement in the pathogenesis of NMO.

Basic structural pathology of the spinal cord in NMO typically shows extensive demyelination across multiple levels associated with necrosis, cavitation, and axonal loss, involving both the gray and white matter. Lesions typically localize to the central cord, and there is evidence of myelin preservation in the periphery, with a significant loss of oligodendrocytes. In chronic lesions, evidence of gliosis, atrophy, and cavitation is commonly seen. The optic nerves and chiasm have also been reported to show inactive demyelination, partial remyelination, as well as gliosis and cavitation [56, 57].

Nagelhus et al. described two distinct types of NMO lesions: those with AQP4 loss and demyelination and necrosis, which are typically seen in the optic nerve and spinal cord, and those with AQP4 loss without demyelination or necrosis, which are typically seen in the medulla and area postrema [49]. The absence of demyelination and necrosis in the latter type of lesions corresponds to the reversible nature of MRI lesions in the medulla and area postrema, as well as resolution of clinical dysfunction attributable to these regions [5].

Inflammatory infiltrates within active demyelinating lesions consist of extensive infiltration of macrophages and microglia, numerous B lymphocytes, occasional CD3+ and CD8+ T lymphocytes, as well as prominent perivascular granulocytes and eosinophils. In early active demyelinating lesions, there is prominent IgG and complement reactivity and macrophage staining in a distinct perivascular rosette pattern, as well as along the rim of thickened vessel walls, suggesting a role for humoral autoimmunity in NMO disease mechanisms [56–58].

Accumulating pathological evidence is convincing that the targeted attack of AQP4 by the NMO-IgG is an important initiating event in the development of NMO. The discovery of a disease-specific autoantibody in NMO, the identification of AQP4 as the antibody target [40, 41], and the demonstration that most NMO lesions have marked loss of AQP4 provide support for this hypothesis [49]. Furthermore, in NMO lesions, the pattern of AQP4 loss has been shown to correspond to the characteristic vasculocentric rim and rosette pattern of IgG and complement deposition. Finally, areas with marked AQP4 loss concurrently show significantly reduced staining for glial fibrillary astrocytic protein (GFAP), with relatively preserved myelin-basic protein, suggesting that astrocytes are the primary target of the NMO-IgG [58]. Taken together, these pathological findings support the notion that a targeted attack of AQP4 in astrocytic foot processes plays a prominent role in NMO pathogenesis involving an immune cascade of events which ultimately results in the clinical manifestations of the disease.

Different hypotheses on the exact immunopathogenic mechanisms by which NMO-IgG exerts it effects have been promoted by separate groups. Lucchinetti et al. have postulated that binding of NMO-IgG to AQP4 initiates two separate events: activation of the lytic complement cascade, and downregulation of AQP4 by endocytosis and degradation [56, 59–61]. A few important observations support this notion, including: the colocalization of immunoglobulin with a marker of the terminal lytic complement complex (C9neo antigen), the presence of activated macrophages and significant vascular hyalinization in perivascular regions in active NMO lesions [56], and in vitro experiments which demonstrate the colocalization of AQP4 with EAAT2, a glutamate transporter and the fact that AQP4 and EAAT2 were endocytosed in the presence of NMO-IgG [59]. Based on these observations, Hu and Lucchinetti propose that binding of NMO-IgG to AQP4 in astrocytic foot processes initiates complement-mediated effects but also disrupts glutamate homeostasis, which can lead to oligodendrocyte injury in the spinal cord and optic nerves, both of which are highly sensitive structures to fluctuations in ambient glutamate concentrations [61]. In addition, they postulate that aberrations in glutamate homeostasis render these structures more sensitive to complement-mediated attacks. On a similar note, Misu et al. found significantly diminished AQP4 and GFAP staining, most prominently in perivascular regions with complement and immunoglobulin deposition with relative preservation of myelin-basic protein. The conclusion from this group was that these observations provide further support for the hypothesis that astrocytic impairment associated with humoral autoimmunity directed against AQP4, which causes AQP4 downregulation, is the primary immunopathogenic mechanism in NMO [58].

By way of comparison, Parratt and Prineas demonstrated in pathological case series examining NMO versus MS lesions that the chief pathological feature unique to NMO was not downregulation of AQP4 expression, but an early complete destruction of perivascular astrocytes, with gliosis initiated by a population of astrocyte progenitors [62]. Although this conclusion can still support an important pathogenic role of NMO-IgG and complement-mediated damage to astrocytes in NMO, it raises the possibility that the acute breakdown of large numbers of astrocytes may in fact be the initiating factor for the generation of NMO-IgG, rather than vice versa.

More recently, in a pathological case series of NMO versus MS lesions, Matsuoka et al. found significant variability in the degree of AQP4 loss in actively demyelinating lesions in between patients with NMO and significant lesion-to-lesion heterogeneity of AQP4 expression even in patients with preferential AQP4 loss. The authors also observed significant variability in the relationship between AQP4 loss and the perivascular deposition of activated complement and immunoglobulin. Furthermore, different patterns of AQP4 loss and perivascular deposition of activated complement and immunoglobulins were observed even within a single lesion. The conclusion from this study was that that there is a heterogeneous relationship between anti-AQP4 antibody and loss of AQP4 expression and that AQP4-mediated immunological destruction may not be the sole mechanism by which NMO lesions are formed [63].

Animal studies have shed some insight into the complexity of NMO disease mechanisms by demonstrating that the presence of the NMO-IgG alone is not directly pathogenic. Kinoshita et al. demonstrated that while passive transfer of IgG obtained from NMO patients to EAE-induced Lewis rats resulted in active lesions with histopathological features suggestive of those observed in human NMO [64], a subsequent study by Bradl et al. showed that the infusion of IgG from NMO patients to healthy (i.e., non-EAE) rats does not cause lesions or clinical symptoms suggestive of human NMO [65]. These findings suggest that human NMO-IgG in and of itself is not pathogenic in rodents but requires the presence of T-cell-mediated CNS inflammation to exert its pathogenic effects. Clinical reports of NMO-IgG identified in patients many years before the clinical onset of symptoms [66, 67] and a significant portion of patients reporting antecedent viral infections prior to symptom onset are both supportive of this hypothesis [3]. It is worth noting, however, that the pathogenic effects of human NMO-IgG infused into these EAE mice may have been exaggerated due to the species-specific nature of complement inhibitors, which are typically abundant throughout the CNS.

In addition to T-cell-mediated CNS inflammation, a complex array of immune cells are evidently at play in NMO disease pathogenesis. Recently, Chihara et al. demonstrated that a subpopulation of B cells resembling plasmablasts was increased in the peripheral blood of patients with NMO and that this population of cells was predominately responsible for the production of NMO-IgG. Interestingly, IL-6 was found to increase the survival of this population of plasmablasts and blockade of IL-6 receptor signaling reduced their survival. From a therapeutic perspective, this is of significant interest in NMO as IL-6 receptor blockading agents (tocilizumab) are already approved for clinical use in the treatment of other autoimmune disorders such as rheumatoid arthritis [68].

To date, an animal model that recapitulates all aspects of human NMO has yet to be identified. The development of such a model of disease will enable further elucidation of proposed disease mechanisms. Until then, we will continue to use various animal models including those that are based on EAE with passive transfer of NMO-IgG.

Taking all of the aforementioned pathological, in vitro, and animal studies into account, it is evident that NMO-IgG plays an important role in NMO disease mechanisms, However, at present, it is difficult to derive any definitive conclusions on the specific role of AQP4 in evolving NMO lesions. The development of more accurate animal models of NMO and further pathological studies in humans will allow clarification of the precise sequence of pathological events in NMO.

## 9. Treatment

### 9.1. Treatment of Acute Relapses

Acute relapses of NMO are generally treated with high-dose intravenous methylprednisolone at 1 g daily for 3–5 days. In some cases, this is followed by an oral prednisone taper. This practice is based on evidence from MS treatment trials rather than any specific controlled trials in NMO patients. Observational studies have shown that the majority of patients (80%) improve with a short course of methylprednisolone [3].

The efficacy of PLEX has been evaluated in a series of retrospective studies which demonstrated clinical improvement in 50–60% of steroid-refractory NMO patients who were treated with PLEX [69, 70], as well as significant clinical improvement in PLEX-treated patients in comparison to those treated with steroids alone [71]. A randomized, sham-controlled trial demonstrated the efficacy of PLEX in a combination of acute CNS demyelinating diseases, which included 2 cases of NMO. The proportion of patients with clinical improvement was significantly higher in patients who had undergone PLEX in comparison to sham treatment (42.1% versus 5.9%) [72]. The typical courses of PLEX used in these reports consisted of 4–7 exchanges over a period of 5–14 days.

The literature supporting the use of IVIG in NMO relapses is sparse and includes only one isolated case report [73]. Therefore, there is insufficient evidence to support the utility of IVIG in the treatment of acute relapses of NMO.

### 9.2. Preventive Treatment

The preventive treatments used in NMO are based largely on retrospective or open-label trials and case series, which makes it difficult to draw definitive conclusions on the efficacy of many of these agents. A summary of the existing evidence for various preventive agents is presented below.

#### 9.2.1. Immunomodulatory Agents

The use of IFN-beta-1b in the preventive treatment of NMO has been reported predominately in the context of Japanese OSMS, and most reports support the notion that interferons are not helpful and may even be harmful in these patients. Uzawa et al. compared the efficacy of IFN-beta-1b in NMO versus MS patients and found that this agent was ineffective in NMO patients [74]. Warabi et al. reported clinical relapses consisting of optic neuritis and myelitis in patients with OSMS treated with IFN-beta-1b [75]. Shimizu et al. reported the development of tumefactive brain lesions in 2 NMO patients treated with IFN-beta-1b, with complete clinical stabilization after the initiation of immunosuppressive treatment with azathioprine [76]. More recently, in a retrospective analysis of carefully selected patients with “genuine” OSMS (rather than NMO), Shimizu et al. reported a significant therapeutic benefit of IFN-beta-1b, with diminished relapse rates and disability progression [77]. In a retrospective analysis conducted by Papeix et al. comparing NMO patients treated with various immunosuppressive agents versus interferon-beta treatment, those treated with immunosuppressive agents showed a significant decrease in relapse rate [78]. A recent case report by Palace et al. reported clinical relapses and high anti-AQP4 Ab titres in a patient with NMO treated with interferon-beta-1a and subsequent clinical stabilization and lower titres with the initiation of azathioprine [79]. Taken together, the existing literature does not support the use of interferon-beta immunomodulatory treatment in NMO and even suggests that these agents may exacerbate the disorder. An important caveat to this conclusion, however, is that, in some cases of “genuine” OSMS, IFN-beta-1b may have therapeutic efficacy.

On the other hand, the use of Copaxone, another first-line immunomodulatory agent used in MS, has been reported in the context of NMO in only two case reports, both which showed a beneficial response [80, 81].

#### 9.2.2. Immunosuppressive Agents

The humoral-mediated immune mechanisms underlying NMO provide theoretical support for the use of rituximab, which is a monoclonal antibody targeting CD20, a protein present on the surface of mature B-lymphocytes. The role of rituximab in ameliorating NMO disease mechanisms by B-lymphocyte depletion has yet to be definitely clarified, but recent evidence demonstrating that CD20-negative plasmablasts are the main subgroup of B-cells responsible for the production of NMO-IgG [68] suggests that rituximab acts through mechanisms other than diminishing the production of antibodies, and possibly via antigen-presenting cells [82]. The efficacy of rituximab in NMO was initially demonstrated in an open-label trial by Cree et al. where 8 NMO patients were treated and followed for a mean of 12 months. The majority (75%) of patients remained relapse-free at follow-up, and all but one patient (87.5%) showed significant neurological improvement. Rituximab was well tolerated in this trial, and no patients experienced any serious adverse reactions [82]. A subsequent retrospective multicenter case series of 25 patients by Jacob et al. showed a significant decrease in relapse rate at a median follow-up of 19 months, as well as disability stabilization or improvement in the majority of patients (80%). Of note, 20% of patients in this series developed new or reactivated infections, and 1 patient died of sepsis [83]. In support of the safety profile of this agent, the literature on the use of rituximab in hematological and rheumatological disorders confirms the long-term safety of this agent, with serious adverse effects seen in only a small minority of patients [84]. A recent report by the European Federation of Neurological Societies by Zhang and Verkaman recommends rituximab as a first-line agent [55], which has also been our practice at Johns Hopkins.

The optimal dosing regimen of rituximab has yet to be determined, and existing studies have utilized differing dosing regimens. Our practice at Johns Hopkins has been to give a single dose of rituximab at 1000 mg intravenously initially, followed by a repeat dose of 1000 mg intravenously after two weeks. Thereafter, CD19/20 cell counts are monitored on a monthly basis. When the CD19/20 cell counts climb above 0.1% of the total lymphocyte count, patients are given the same double dose of rituximab (1000 mg iv × 1, followed by 1000 mg iv after two weeks). If the CD19/20 cell count is undetectable 6 months after the last dose, a single dose of 1000 mg iv is given, and CD19/20 counts are followed closely. In children, an initial single dose of rituximab of 375 mg/m^2^ is given, and this dose is repeated when the CD19/20 cell count climbs above 0.1% as in adults. Further laboratory parameters to routinely monitor are outlined in Table 1.

Azathioprine (AZA) is an oral immunosuppressive agent commonly used in organ-transplant-related immunosuppression, as well as in the treatment of systemic autoimmune disorders such as SLE and rheumatoid arthritis. AZA is a purine antagonist that interferes with DNA and RNA synthesis, which results in immune cell inhibition by a variety of mechanisms. The efficacy of AZA in the preventive treatment of NMO patients was described by Mandler et al. in an open-label prospective trial of 7 NMO patients, where sustained disability improvement and the absence of any subsequent relapses over 19 months was documented in all subjects [85]. Subsequently, Bichuetti et al. performed a retrospective review of 36 cases of NMO, of which 27 patients were on AZA at a mean follow-up of 47 months. AZA alone or in combination with prednisone was shown to significantly diminish relapse rate and stabilize disability [86]. Of note, in both reports, there were no serious adverse effects in any patients treated with AZA. Most recently, Costanzi et al. performed a retrospective review of 99 patients with NMO spectrum disorders treated with AZA. AZA used alone or in combination with prednisone significantly reduced annualized relapse rates by 76%, and in 70 patients with more than 12 months of follow-up EDSS was stable or improved in 60% of patients. Of note, 3 cases of lymphoma were reported in this study [87]. The causal link between AZA and lymphoma is at present controversial; however, until further information is available, this is another serious adverse effect of which clinicians should be aware. The utility of AZA in diminishing relapse rate has also been documented in a handful of case reports and case series [76, 78], as well as a subset of a cohort of NMO patients described by Jarius et al. [88].

The dosing regimen for AZA varies across different centers, but at Johns Hopkins, our preference is to start at 2 mg/kg/day, divided into two daily doses. If there is no clinical response, the dose is increased to 3 mg/kg/day. For resistant cases, prednisone at 1 mg/kg/day is added or the decision is made to switch to another immunosuppressive agent. Patients considering initiating AZA should be tested for the thiopurine S-methyltransferase (TPMT) mutation, which is seen in up to 10% of the US population [89]. Individuals harboring this mutation can develop significant bone marrow toxicity with AZA; therefore, this medication is best avoided in these patients. Further laboratory parameters to monitor are outlined in Table 1.

Mycophenolate mofetil (MMF) is another oral immunosuppressive agent commonly used in organ-transplant-related immunosuppression and rheumatological disorders. MMF acts as an inhibitor of a rate-limiting enzyme in the synthesis of guanine ribonucleotide and 2-deoxyribonucleotide, which ultimately suppresses the immune system by inhibiting dendritic cell and T and B lymphocyte functioning. The efficacy of MMF in NMO was described in a retrospective case series of 24 patients with NMO and NMO spectrum disorders, which demonstrated a significantly diminished relapse rate, as well as stable or improved disability measures in the majority (91%) of patients which included rituximab failures. Of note, 25% of patients reported adverse effects, and 1 patient had a low WBC count that necessitated discontinuing the medication [90]. One serious adverse effect derived from the transplant literature is a 14.4 in 100,000 risk of PML [91].

At Johns Hopkins, our practice has been to utilize MMF as a first-line agent. Patients are started on a dose of 500 mg twice daily, which is titrated up to 1000 mg twice daily over 4 weeks. At the 4-week mark, the WBC count and differential is assessed. The target total WBC count is 3-4 × 10^3^/*μ*L (approximately half of normal). Alternatively, the target absolute lymphocyte count is 1-1.2 × 10^3^/*μ*L. The absolute lymphocyte count should not fall below 1 × 10^3^/*μ*L. If either target is not attained, the MMF dose is increased by 250 mg twice daily every two weeks, up to a maximum of 1500 mg twice daily. If the target is not attained at the maximum 1500 mg twice daily, other immunosuppressive agents such as rituximab can be considered. While the MMF dose is being titrated up to the target dose, prednisone is typically co-administered at 20–30 mg daily and weaned off when the target MMF dose is reached. Monthly liver function tests should be performed to rule out toxicity for six consecutive months while the dose is titrated, then twice yearly thereafter on a stable dose. Further laboratory parameters to monitor are outlined in Table 1.

Cyclophosphamide (CYC) is an intravenous immunosuppressive drug that acts as a DNA alkylating agent, which ultimately impairs T and B lymphocyte activity, as well as various inflammatory cytokines. CYC is used in combination with other agents in the treatment of various malignancies, as well as in the treatment of systemic autoimmune disorders. There are no extensive case series or controlled trials on the use of CYC in NMO, but isolated case reports have shown a beneficial clinical effect on relapse rate and disability in patients with isolated NMO [73, 88], as well as in patients with NMO/SLE overlap [92, 93].

Methotrexate (MTX) is a dihydrofolate reductase inhibitor and acts by impairing DNA synthesis. MTX is a commonly used drug in the treatment of systemic autoimmune disorders and in combination with other drugs as a chemotherapeutic agent. The utility of a combination of MTX and prednisolone in NMO was described in a case report by Palace et al., where a patient remained relapse-free 3 years after the initiating treatment [79].

Mitoxantrone (MITO) is a synthetic anthracenedione derivative that acts by inhibiting both DNA and RNA synthesis, which results in suppression of both T and B lymphocytes, and inhibition of proinflammatory cytokine secretion. The efficacy of MITO in NMO was demonstrated in a prospective open-label two-year study where 4 out of 5 patients (80%) showed significant clinical and radiographic improvement. In this study, MITO was generally well tolerated, with one patient having evidence of sub-clinical cardiac insufficiency that recovered after medication discontinuation [94]. A subsequent retrospective case series by Kim et al. showed that MITO resulted in a significant decrease in relapse rate and stabilization or improvement in measures of disability in all patients. There were no serious adverse effects in any patients [95]. Despite the apparent tolerability of MITO in these two trials, experience in MS and other disorders has shown that MITO can causes serious toxicity with reports of potentially lethal adverse effects such as opportunistic infection, cardiac systolic dysfunction, and therapy-related acute leukemia [96]. Therefore, the clinical benefits associated with the use of MITO in NMO must be carefully weighed against the potential serious risks and the fact that the long-term tolerability in NMO patients remains unknown.

Case reports have suggested that intermittent IVIG infusions may be of utility in preventing relapses and clinical deterioration. Bakker and Metz reported 2 cases of NMO treated with monthly infusions of IVIG that resulted in a significant decrease in relapse rate and clinical improvement [97]. More recently, Okada et al. report a case of NMO that responded with monthly IVIG with respect to relapse rate and clinical disability [98]. A recent case series of 2 patients with treatment-refractory NMO showed a significant decrease in relapse rate with the initiation of intermittent plasmapheresis [99]. Although the existing evidence is not sufficient to recommend intermittent IVIG as preventive treatment in NMO, it may be considered for research trials in treatment-refractory individuals.

## 10. Conclusion

In the past decade, the discovery of a disease-specific antibody has enabled the establishment of NMO as a distinct disease entity in the spectrum of CNS demyelinating disorders. What was once thought of as a variant of MS is now regarded as a unique disease entity with significantly different prognostic and treatment implications.

Although much progress has been made in clarifying the clinical, epidemiological, radiographic, and pathological features of NMO, much remains to be understood. Future directions of investigation include the development of an accurate animal model of disease, refinement of immunoassays for detection of the NMO-IgG, application of novel imaging techniques, and clarification of the epidemiological and genetic risk factors of NMO. Together, this will enable earlier disease detection and, ultimately, the development of more targeted treatment strategies for this debilitating neurological disorder.

## Figures and Tables

**Table 1:** Recommended first-line agents in the preventive treatment of NMO.

Medication	Initial dosing Regimen	Maintenance dosing regimen	Monitoring guidelines
Azathioprine (AZA)	2 mg/kg/day p.o., divided into 2 daily doses	(i) Increase to 3 mg/kg/day p.o. if unsatisfactory response (ii) Add prednisone 20–30 mg p.o. daily if unsatisfactory response (iii) Switch to alternate immunosuppressive agent if unsatisfactory response	(i) TPMT genotyping: avoid use in TPMT positive patients (ii) CBC and differential baseline and qweekly × 4, qbiweekly × 2, then q1-2 months, LFTs q3months

Mycophenolate mofetil (MMF)	500 mg p.o. bid	(i) Titrate up to 1000 mg p.o, bid over 4 weeks (ii) Check WBC count and differential at 4 weeks: target total WBC = 3-4 × 10^3^/*μ*L or absolute lymphocyte count =1–1.2 × 10^3^/*μ*L (iii) If target WBC or lymphocyte count unattained, increase dose by 250 mg p.o. bid every 2 weeks to maximum dose of 1500 mg p.o. bid (iv) Administer prednisone at 20–30 mg p.o. daily while titrating up MMF, wean off prednisone over 6–8 weeks once target dose attained (v) Switch to alternate immunosuppressive agent if target WBC/lymphocyte count not attained or unsatisfactory clinical response	(i) CBC and differential at baseline and qweekly × 4, then qmonthly × 6 months, then q6monthly (ii) LFTs at baseline and qmonthly × 6 months, then q6monthly (iii) If clinical suspicious for infection: septic work-up, including CSF JC virus if suspicious for PML

Rituximab	1000 mg iv × 1 dose	(i) Repeat 1000 mg iv × 1 dose 2 weeks after initial dose (ii) Check CD19/20 cell counts monthly (iii) Redose with same double dose (1000 mg iv × 1, followed by repeat dose 2 weeks later) when CD19/20 cell counts > 0.1% total lymphocyte count (iv) If CD19/20 counts are undetectable 6 months after last dose, redose with single 1000 mg iv dose	(i) Baseline and monthly CBC and differential, CD19/20 cell count (ii) Baseline and periodic renal function tests (iii) Screen for hepatitis B in high-risk patients prior to initiation
